# The Academic Motivation and Engagement of Students in English as a Foreign Language Classes: Does Teacher Praise Matter?

**DOI:** 10.3389/fpsyg.2021.778174

**Published:** 2021-10-28

**Authors:** Chunju Peng

**Affiliations:** ^1^English Department, Hainan Medical University, Haikou, China; ^2^Department of Postgraduate Studies, Faculty of Education, Languages and Psychology, SEGi University, Kota Damansara, Malaysia

**Keywords:** teacher praise, academic motivation, academic engagement, EFL classes, positive academic emotion

## Abstract

Given the undeniable role of English as a foreign language (EFL) students’ academic motivation and engagement in L2 success, identifying the antecedents of these positive academic behaviors seems essential. Accordingly, many empirical studies have probed into the impact of students’ personal factors on their motivation and engagement. Yet, not much attention has been paid to the role of teachers’ communication behaviors, notably praise. Additionally, no review has been performed in this regard. The present review study intends to address these gaps by explaining teacher praise and its positive outcomes for EFL students’ motivation and engagement. In light of the empirical and theoretical evidence, the role of teacher praise in improving students’ academic motivation and engagement was proved. The paper concludes with some pedagogical implications.

## Introduction

Students’ motivation and engagement as two prime instances of positive academic behaviors serve a facilitative function in their learning success (Martin et al., 2017). Accordingly, raising students’ academic motivation and engagement has been among the top priorities of all effective instructors. However, many instructors, notably English as a Foreign Language (EFL) and English as a Second Language (ESL) teachers are still unaware of how to considerably enhance their students’ academic motivation and engagement. In fact, how EFL and ESL students’ academic motivation and engagement can be improved is not widely recognized (Henry and Thorsen, 2018). Students’ academic motivation or motivation to learn generally refers to “their primary impetus for initiating learning as well as the reason for continuing the prolonged and tedious process of learning” (Ushioda, 2008, p. 21). More specifically, conceptualized language learners’ academic motivation as the degree to which they strive to acquire a new language out of a desire to do so and the enjoyment they experienced in the process of learning. Besides, student academic engagement as another example of desirable academic behaviors pertains to “students’ active, goal-directed, flexible, constructive, persistent, focused interactions with the learning environment” (Furrer and Skinner, 2003, p. 149). In the domain of language education, students’ academic engagement refers to their active participation in learning and mastering a new language (Hiver et al., 2021).

As Irvin et al. (2007) noted academic motivation and engagement as two related constructs are of high importance for students’ increased achievement, advancement, and academic success. Concerning the value of student academic motivation in instructional-learning environments, Froiland and Oros (2014) postulated that both intrinsic and extrinsic motivation of pupils can favorably influence their academic performance. In a similar vein, Martin (2013) also stated that the sense of enjoyment that highly motivated students experience in classroom contexts encourages them to enthusiastically pursue different stages of learning. This, in turn, contributes to desirable learning outcomes. In this regard, Howard et al. (2021) also illustrated the importance of motivation by referring to its positive effect on students’ level of perseverance. They articulated that academic motives can empower students to resist the difficulties that they may experience during the learning process.

To shed light on the significance of student academic engagement, Skinner and Pitzer (2012) mentioned that students’ active membership in instructional-learning contexts enables them to gain higher academic grades. Similarly, Finn and Zimmer (2012) submitted that students’ degree of participation in educational contexts is closely related to their academic growth. To them, nothing like active participation in classrooms can facilitate students’ educational advancement. Additionally, Philp and Duchesne (2016) also postulated that students’ academic engagement can remarkably increase the likelihood of their academic success. Drawing on what has been mentioned regarding the centrality of academic motivation and engagement in students’ educational success, investigating the determinants and predictors of these variables seems crucial.

Against this backdrop, numerous studies have inspected the impact of students’ personality traits on their academic motivation (e.g., Komarraju et al., 2009; De Feyter et al., 2012; Hazrati-Viari et al., 2012; Zhang et al., 2013; Guo, 2021) and engagement (e.g., Linvill, 2014; Kahu et al., 2015; Qureshi et al., 2016; Rostami et al., 2017). Several studies have also been conducted to examine the effects of teachers’ personality traits on students’ motivation and engagement (e.g., Gibbs and Powell, 2012; Sabet et al., 2018; Khalilzadeh and Khodi, 2021). Additionally, many empirical and theoretical studies have probed into the role of teachers’ positive communication behaviors, including credibility (e.g., Imlawi et al., 2015; Derakhshan, 2021), immediacy (e.g., Dixson et al., 2017; Liu, 2021; Zheng, 2021), and confirmation (e.g., Shen and Croucher, 2018; LaBelle and Johnson, 2020; Gao, 2021), in promoting student academic motivation and engagement. Nonetheless, teacher praise as one of the most influential communication behaviors has received scant attention (Downs, 2017; Caldarella et al., 2021).

The concept of praise generally refers to “verbal or nonverbal actions indicating the positive quality of a behavior over and above the evaluation of accuracy” (Kalis et al., 2007, p. 23). Similarly, teacher praise pertains to any gesture or statement that instructors employ to admire their students’ appropriate and favorable behaviors (Reinke et al., 2008). Jenkins et al. (2015) postulated that teacher praise is a “*feasible, nonintrusive classroom strategy*” that can be easily utilized by teachers in any instructional-learning environment. As Marchant and Anderson (2012) noted, the verbal or nonverbal action used by teachers to applaud their pupils’ positive behaviors may work as a stimulator, encouraging students to repeat the desired actions. They also suggested that teachers can inspire a feeling of mastery and accomplishment in their pupils by acknowledging their satisfactory behaviors. These positive feelings contribute to increased student motivation (Titsworth, 2000). According to Richard (2012), students’ willingness to participate in classroom activities also improves when instructors praise their academic performance.

Despite the importance of teachers’ verbal and nonverbal admiration in increasing student’ academic motivation and engagement (Marchant and Anderson, 2012; Richard, 2012), a few scholars (Downs, 2017; Caldarella et al., 2021) have studied teacher praise in relation to these positive academic behaviors. Furthermore, no theoretical or systematic review has been carried out in this regard. Thus, to narrow the existing gaps, the current review study aims to provide a detailed description of these variables (i.e., teacher praise, student motivation, and student engagement), their theoretical foundations, and the existing association among them.

## Related Literature

### Teacher Praise

The term praise comes from a Latin verb, namely “*pretiare*,” which means “to value highly” (Burnett, 2002, p. 6). This construct is literally defined as “the expression of approval or admiration for one’s behavior or characteristic” (Brophy, 1981, p. 5). In line with this definition, Burnett and Mandel (2010) conceptualized teacher praise as positive verbal or nonverbal actions through which teachers glorify students whenever they perform well. As clearly mentioned in this definition, like other communication behaviors such as immediacy, confirmation, and stroke (Han and Wang, 2021; Xie and Derakhshan, 2021), teacher praise can be both verbal and nonverbal. As Shernoff et al. (2020) noted, verbal praise refers to any positive comments that teachers offer to students due to their desired academic behaviors. Nonverbal praise also pertains to any gestures, including nodding and smiling, teachers use to exalt their pupils. Generally, teacher praise is of two types: “*General Praise (GP)*” and “*Behavior-Specific Praise (BSP)*” (Floress et al., 2017). GP means admiring students’ behavior without mentioning which aspects of their performance were acceptable (Duchaine et al., 2011). In contrast, BSP, as the name speaks for itself, entails “approval with an explanation of the appropriate behavior exhibited” (Duchaine et al., 2011, p. 210).

### Student Academic Motivation

The concept of motivation is generally conceptualized as a stimulating force that directs human behaviors (Brophy, 1983). Student motivation to learn, also known as academic motivation, is related to their motive “to make certain academic decisions, participate in classroom activities, and persist in pursuing the demanding process of learning” (Dörnyei and Ushioda, 2009, p. 2). Working on different types of student academic motivation, Brophy (1983) divided this construct into two broad categories, namely “*state motivation*” and “*trait motivation*.” State motivation refers to “students’ attitude toward a particular course” (Guilloteaux and Dörnyei, 2008, p. 56). Trait motivation, on the other hand, deals with students’ general tendency toward the learning process (Csizér and Dörnyei, 2005). While students’ trait motivation is typically constant during a whole course, their state motivation is open to drastic changes (Trad et al., 2014). As Hiver and Al-Hoorie (2020) mentioned, student state motivation can be dramatically influenced by their viewpoints and attitudes toward their instructors, course content, and learning environment. Similarly, Dörnyei (2020) also posited that how students perceive their teachers’ personal and interpersonal behaviors has a significant impact on their academic motivation. It implies that those teachers who behave appropriately in classroom contexts have a beneficial impact on their students’ state motivation (Cheng and Dörnyei, 2007; Bernaus and Gardner, 2008; Papi and Abdollahzadeh, 2012).

### Student Academic Engagement

Student engagement, in a general sense, refers to the amount of time, energy, and effort that students willingly dedicate to educational activities (Appleton et al., 2008). Skinner et al. (2009, p. 495) conceptualized this construct as “the quality and quantity of students’ participation or connection with the educational endeavor and hence with activities, values, individuals, aims, and place that comprise it.” Despite the existing controversy regarding the terminology of this concept, many scholars referred to this construct as “*student academic engagement*” (e.g., Leach and Dolan, 1985; Greenwood et al., 2002; Brint et al., 2008). Other academics named this construct as “*school engagement*” (Jimerson et al., 2003), “*educational engagement*” (Wehlage et al., 1989), and “*study engagement*” (Schaufeli et al., 2002). Similarly, there has been a long debate over the number and types of the components of this construct (Alrashidi et al., 2016). As an instance, Audas and Willms (2001) classified the components of student engagement into two broad categories, whereas Fredricks et al. (2004) divided this construct into three main dimensions (Table 1).

**Table 1:** Different dimensions of academic engagement.

Authors	Dimensions
Audas and Willms, 2001	Behavioral engagement: Taking part in educational tasks and activities Psychological engagement: Having a sense of identification, belonging, and inclusion in the learning context
Fredricks et al., 2004	Behavioral engagement: Active participation in learning activities Emotional engagement: Positive disposition toward instructors, classmates, and the learning environment Cognitive engagement: Mental efforts to learn the difficult and challenging content

Despite all the aforementioned discrepancies, researchers have come to the conclusion that the construct of academic engagement is multidimensional and covers several aspects, including cognitive, emotional, and behavioral, working together to demonstrate students’ positive attitudes toward the learning process (Hiver et al., 2021).

### The Impact of Teacher Praise on EFL Students’ Academic Motivation and Engagement

The impact of teacher praise on EFL students’ level of motivation and engagement can be readily illustrated through “*Emotional Response Theory (ERT)*.” In their theory, Mottet et al. (2006) asserted that the positive communication behaviors, including praise, used by language teachers while instructing may result in learners’ positive responses such as happiness, L2 enjoyment, and pleasure. To them, those language learners who experience a sense of happiness, pleasure, or enjoyment in the learning environment are more motivated to pursue the learning process. Those who are sufficiently motivated tend to actively take part in classroom activities (Martin, 2007; Pekrun and Linnenbrink-Garcia, 2012; Reeve, 2012). In a similar vein, drawing on the positive psychology movement (Dewaele et al., 2019; MacIntyre et al., 2019; Wang et al., 2021), Xie and Derakhshan (2021) also illustrated the favorable association between teacher communication behaviors and students’ academic behaviors (e.g., engagement, motivation, etc.). They stated that, through effective communication behaviors, language teachers are able to create a pleasant educational atmosphere, wherein learners enjoy learning a new language. Having a sense of enjoyment is of high importance for students’ increased motivation and engagement (Kulakow and Raufelder, 2020; Pedler et al., 2021). All in all, based on what Mottet et al. (2006) and Xie and Derakhshan (2021) mentioned, teacher praise as an instance of effective communication behavior can considerably influence EFL students’ engagement and motivation.

A number of empirical studies have shed light on the extent to which teacher praise is linked to students’ academic motivation and engagement (Richard, 2012; Downs, 2017; Caldarella et al., 2021). As an instance, Richard (2012) examined the impact of teachers’ verbal and nonverbal praise on students’ engagement. In doing so, a group of American teachers and students took part in this inquiry. Some treatment sessions were run to observe the effects of teacher verbal and nonverbal praise on students’ classroom engagement. The analysis of the obtained data demonstrated that students’ participation in classroom exercises was positively influenced by their teachers’ verbal and nonverbal praise. In another study, Downs (2017) probed into the effects of teacher praise on student’ emotional behaviors, namely motivation and engagement. To this aim, 239 students were invited to attend some treatment session. The results of observations indicated that the verbal and nonverbal praise that teachers provided in treatment sessions favorably affected participants’ motivation and engagement.

## Conclusion and Pedagogical Implications

So far, various definitions of student academic motivation, academic engagement, and teacher praise, along with their theoretical foundations, were illustrated. Building upon emotional response theory and the positive psychology movement, the association between these variables was also explained. Additionally, a summary of the previous related studies was provided. Based on what has been reviewed in the current study, it is fair to conclude that teacher praise (verbal or nonverbal) is a strong antecedent of EFL students’ academic motivation and engagement. This finding can be highly beneficial for all EFL teachers who struggle with their students’ insufficient motivation and engagement. As noted by Mottet et al. (2006) and Xie and Derakhshan (2021), through admiring students’ behaviors, teacher can dramatically enhance student’ engagement and motivation to learn. The review’s finding has an important implication for teacher trainers as well. An important reason underlying EFL students’ lack of motivation and engagement is teachers’ disability to praise students’ behaviors (Duchaine et al., 2011). Thus, to improve EFL students’ motivation and engagement, teachers should receive adequate instructions on how to praise their students’ academic performances. Put it simply, teacher trainers should teach EFL instructors all they need to know regarding this positive communication behavior.

## Author Contributions

The author confirms being the sole contributor of this work and has approved it for publication.

## Funding

This paper is a special subject of Hainan Philosophy and Social Sciences Foreign Language Application Research Base “Study on Cultivation of Intercultural Communication Competence of College Students in Hainan Medical College under the Background of Free Trade Port” (grant no. HNWYJD18-05).

## Conflict of Interest

The author declares that the research was conducted in the absence of any commercial or financial relationships that could be construed as a potential conflict of interest.

## Publisher’s Note

All claims expressed in this article are solely those of the authors and do not necessarily represent those of their affiliated organizations, or those of the publisher, the editors and the reviewers. Any product that may be evaluated in this article, or claim that may be made by its manufacturer, is not guaranteed or endorsed by the publisher.
